# Dysphagia in Amyotrophic Lateral Sclerosis: Impact on Patient Behavior, Diet Adaptation, and Riluzole Management

**DOI:** 10.3389/fneur.2017.00094

**Published:** 2017-03-21

**Authors:** Emanuela Onesti, Ilenia Schettino, Maria Cristina Gori, Vittorio Frasca, Marco Ceccanti, Chiara Cambieri, Giovanni Ruoppolo, Maurizio Inghilleri

**Affiliations:** ^1^Rare Neuromuscular Diseases Centre, Department of Neurology and Psychiatry, Sapienza University, Rome, Italy; ^2^Department of Sensorial Organs, Otorhinolaryngology Section, Sapienza University, Rome, Italy

**Keywords:** amyotrophic lateral sclerosis, dysphagia, riluzole, swallow, diet

## Abstract

This retrospective study aimed to investigate the clinical features associated with deteriorated swallow in amyotrophic lateral sclerosis (ALS) patients with spinal and bulbar onset, describe the modification of diet and liquid intake, and assess the impact of dysphagia on the use of riluzole. One hundred forty-five patients were observed periodically every 3–6 months. They underwent routinely fiberoptic endoscopic evaluation of swallowing (FEES) and spirometry; dysphagia severity was classified according to the Penetration Aspiration Scale and the Pooling score (*P*-score) integrated with other parameters such as sensation, collaboration, and age (P-SCA score). During a mean follow-up period of about 2 years, the percentage of ALS patients suffering from dysphagia increased to 85 (rising from 35 to 73% in patients with spinal onset and from 95 to 98% in those with bulbar onset). Also, 8% of patients with dysphagia by FEES did not perceive the disorder. The frequency of normal and semi-solid diets decreased over time, while that of pureed diets and percutaneous endoscopic gastrostomy (PEG) prescription increased. Forty-four percent of dysphagic patients refused thickeners or PEG. A significant difference was observed in the mortality rate between patients untreated with riluzole and patients treated with riluzole oral suspension (*p* < 0.05). Disease duration mainly impacted on the frequency of dysphagia in spinal onset patients, appearing very early in those with bulbar onset. Riluzole oral suspension would allow the safe administration in dysphagic ALS patients to avoid tablet crushing and consequent dispersion in food, common practices that are inconsistent with the safe and effective use of the drug.

## Introduction

Amyotrophic lateral sclerosis (ALS) is a neurodegenerative disease characterized by a progressive loss of motor neurons that leads to paralysis and death within 2–5 years from the time of diagnosis (1). Generally, death occurs because of respiratory failure, aspiration pneumonia, malnutrition, and dehydration (2).

About one-third of ALS patients show a bulbar onset with dysphagia and dysarthria. Yet, independent of the clinical onset, dysphagia emerges in more than 80% of patients during the advanced phases of the disease (3). In ALS, dysphagia is related to tongue atrophy, dysfunction in the closure of the soft palate and of the larynx due to the nuclear or supranuclear lesion of the cranial nerves, IX, X, and XII, and diaphragm dysfunction (4). A prompt assessment of the swallowing function is crucial to organize proper interventions and prevent rapid clinical deterioration (5). Irrespective of the poor prognosis of ALS, adequate adjustment of the diet and adoption of postural compensation maneuvers to preserve oral feeding could postpone the need for percutaneous endoscopic gastrostomy (PEG) (6, 7). Fiberoptic endoscopic evaluation of swallowing (FEES) and videofluoroscopic swallowing study are both considered the gold standard to evaluate dysphagia (8). However, the endoscopic assessment allows a direct examination of the laryngeal adductor reflex (LAR) and can be repeated as often as necessary due to absence of radiation exposure.

Several methods have been suggested for the treatment of oropharyngeal dysphagia in adults to prevent airway aspiration during feeding (9, 10). The guidelines for the management of ALS recommend PEG when the weight loss exceeds 10% of the baseline value and the forced vital capacity (FVC) decreases below 50% of the predicted level (11).

Physiological swallowing is a crucial parameter also for the proper intake of drugs, and dysphagia can seriously compromise their effect. To date, only riluzole has been approved for the treatment of ALS, even if its clinical usefulness remains controversial (12). Moreover, since 2015, a new oral liquid formulation of riluzole has become available in Italy (Teglutik, Italfarmaco S.p.A.) (13).

Actually, there are no data describing the behavior adopted by the patients because of dysphagia, the impact of swallowing management programs in prolonging survival and in delaying the use of tracheostomy and mechanical ventilation, and the use of riluzole in dysphagic ALS patients.

The primary aim of the present study was to describe the different clinical features associated with dysphagia in ALS patients with spinal and bulbar onset. Furthermore, we aimed to investigate the impact of dysphagia on diet and riluzole intake, analyze the risk of dysphagia in patients unable to recognize disease symptoms, and evaluate the effect of riluzole taken in an uncommon way on survival.

## Materials and Methods

### Study Design and Patients

Since 2005, an ALS registry has been set up at the Rare Neuromuscular Diseases Center of Umberto I Hospital, University of Rome “Sapienza.” All consecutive patients with a probable or confirmed ALS diagnosis were included in the registry. Diagnosis of ALS was based on the El Escorial revised criteria (14, 15). According to the most recent guidelines, patients underwent visits every 3–6 months, receiving regular support from a multidisciplinary care team (16).

In December 2015, we retrospectively conducted an audit of the registry to collect dysphagia data in ALS patients who underwent visits every 3–6 months to analyze dysphagia onset and severity, patient disability, diet modification, and riluzole management. Patients were included in this retrospective study if they had at least three clinical evaluations comprehensive of swallowing assessment (FEES and LAR study) at each visit. Patients with concomitant disorders able to induce dysphagia were excluded.

Participants were divided into two subgroups, bulbar and spinal ALS, according to the clinical onset of disease. These were further classified into dysphagic/non-dysphagic and treated/untreated with riluzole. We defined patients as having a “poor prognosis” when death or survival with tracheostomy occurred during the observational period.

This study was performed according to the Declaration of Helsinki. The authorization to the processing of personal data carried out for scientific research purposes or the written consent for alive patients was provided by all participants.

### Clinical Assessments

At each visit, the following assessments were performed: (i) FEES to evaluate the swallowing function plus completion of the Penetration Aspiration Scale (PAS) and Pooling score (*P*-score); (ii) spirometry to test the respiratory function; and (iii) ALS Functional Rating Scale Revised (ALSFRS-R) to evaluate the functional impairment in ALS.

Fiberoptic endoscopic evaluation of swallowing and LAR, performed by an experienced phoniatrician according to the Langmore protocol (1999), evaluate objectively the swallowing function. A 3.7-mm-diameter flexible fiber-optic rhinolaryngoscope (Storz 11101) was used. The endoscopic evaluation was performed by checking details of pharyngeal and laryngeal surfaces, the competence of the velopharyngeal closure, the morphology, motility, and sensitivity of the larynx, LAR, and secretion residues. The swallowing test consisted in the administration of increasing volumes (i.e., 5, 10, and 20 ml) of puree consistency (351–1,750 mPa s) and liquids.

Penetration Aspiration Scale grades the severity of swallowing dysfunction according to aspiration events (17). It is an 8-point scale quantifying penetration and aspiration in order to clarify the depth of airway invasion. ALS patients were considered dysphagic when the score was >1.

The *P*-score assesses the ability to control residue/bolus pooling (18). It takes into account (i) site, as identified by anatomical landmarks; (ii) amount, as determined in a semiquantitative fashion by the amount of pooling materials; (iii) management, i.e., the ability of the patient to clear the residue (cough, clearing, gurgling, dry swallow). The *P*-score may be integrated with other parameters of clinical assessment: sensation of the pharynx, patient collaboration, and age (P-SCA score) (19) (Table 1). Both scores express, as a numerical value, a continuum of severity that may indicate the absence of signs of dysphagia, mild, moderate, and severe dysphagia (3–4 = minimum score, corresponding to no dysphagia; 5–8 = low score, mild dysphagia; 9–12 = middle score, moderate dysphagia; 13–16 = high score, severe dysphagia).

**Table 1:** **The Pooling score (*P*-score) integrated with parameters as sensation, collaboration, and age (P-SCA score) [by Farneti et al. (8)]**.

Pooling	Endoscopic landmarks	Score	Bedside parameters		
			Sensation	Collaboration	Age (years)
Site	Vallecula	1			
	Marginal zone	1			
	Pyriform sinus	2			
	Vestibule/vocal cords	3			
	Lower vocal cords	4	Presence = −1	Presence = −1	1 (<65)
			Absence = 1	Absence = 1	2 (65–75)
	Coating	1			3 (>75)
Amount	Minimum	2			
	Maximum	3			
Management	<2	2			
	2–5	3			
	>5	4			
Score		*P*: 4–11		P-SCA: 3–16	

The ALSFRS-R is a validated measure of functional impairment in ALS (20). It is a questionnaire-based functional scale, containing 12 items rated from 0 (complete dependence for that function) to 4 (normal function), divided into 3 sub scores (bulbar 12, spinal 24, and respiratory 12), with normal function defined by a score of 48.

Spirometry was carried out with participants in the sitting position wearing a nose clip, asked to blow into the mouthpiece of a spirometer (Winspiro PRO 5.8) as forcefully and quickly as possible and to continue blowing until all of the air was expelled from lungs. FVC (liters) and forced expiratory volume in first second (liters per second) were analyzed. The highest values were used in the analysis. The FVC values were expressed as percentages.

The onset of dysphagic symptoms was reported by patients. The ability to swallow solid food and liquids was assessed by means of anamnestic and clinical criteria. All dietary advice, prescription of PEG, non-invasive ventilation (NIV), changes in pharmacological treatments, use of thickening agents, and adopted diet were collected. Specifically, the patient diet was categorized as described hereinafter: a normal diet without restrictions, a soft diet (>10^12^ Pa s), a pudding diet (1,750–4,000 mPa s), and a pureed diet (351).

The intake of liquid was defined as follows: normal, postural (tucked chin), density 1 (syrup-nectar, 51–350 mPa s; it can be drunk with a straw or a cup, leaves a thin veneer on the back of the spoon), and density 2 (cream, 351–1,750 mPa s; it cannot be drunk with a straw; it can be drunk from a cup; it leaves a thin veneer on the back of the spoon).

The ability to swallow the whole riluzole tablet has been collected within the description of riluzole modification (crushing/swallow with food) in case of inability to swallow.

Patient survival has been calculated from the time of diagnosis until the date of death.

### Statistical Analysis

Descriptive statistics, which summarizes data as percentages, was used. The Student’s *T* distribution was used to evaluate the baseline differences between dysphagic/non-dysphagic patients. The Chi-square test, with Yates’ correction if necessary, was used to compare qualitative values. Analysis of variance (ANOVA) for repeated measures assessed the differences, which were presented as percentage and 95% confidence interval. Statistically significant ANOVA was confirmed by *post hoc* Fisher analysis. Overall survival was analyzed using the Kaplan–Meier method, and *P* values were calculated using the log-rank test and Wilcoxon test. The level of significance was set at 0.05. The data of three assessments (i.e., initial, intermediate, and final visit) were used to measure disease evolution. The survival analysis was performed using Cox Regression.

Generalized linear mixed logit regression models were used to examine the association between death and other variables (age at onset, type of ALS, PAS, and P-SCA scores).

## Results

One hundred and forty five ALS patients (90 males and 55 females) met the inclusion criteria. They were followed for at least 9 months from baseline (mean duration of the clinical follow-up was 20.0 ± 18.3 months; range: 9–110). Mean age at onset was 62.2 ± 11.7 years. The disease duration at the first visit was 15.8 ± 12.7 months (range: 1–78). Fifty-seven patients (39%) had bulbar onset, while 88 patients (61%) had a spinal onset (Table 2).

**Table 2:** **Demographic and clinical characteristics of amyotrophic lateral sclerosis (ALS) patients with or without dysphagia at the start and at the end of follow-up**.

Variable	At the start of follow-up			At the end of follow-up		
	Dysphagic pts	Non-dysphagic pts	*p*	Dysphagic pts	Non-dysphagic pts	*p*
ALS patients *N* (%)	**85 (58.6)**	**60 (41.4)**		**120 (82.8)**	**25 (17.2)**	
Clinical onset *N* (%)						
Bulbar	54 (63.5)	3 (5.0)	0.001	56 (46.7)	1 (4.0)	0.001
Spinal	31 (36.5)	57 (95.0)		64 (53.3)	24 (96.0)	
Age (years) at onset mean (SD)	64.0 (12.3)	59.6	0.02	63.2 (11.9)	57.4 (9.2)	0.02
Sex *N* (%)						
Male	45 (52.9)	45 (75.0)	0.007	75 (62.5)	15 (60.0)	ns
Female	40 (47.1)	15 (25.0)		45 (37.5)	10 (40.0)	
Delay of ALS diagnosis (months) mean (SD)	14.2 (10.7)	18.0 (14.8)	ns	36.1 (23.5)	35.6 (22.0)	ns
Penetration Aspiration Scale mean (SD)	2.4 (1.5)	1.0 (−)	0.001	4.0 (2.0)	1.0 (0.2)	0.001
P-SCA score mean (SD)	6.9 (1.8)	3.1 (0.2)	0.001	9.6 (3.0)	3.4 (0.8)	0.001
ALS Functional Rating Scale Revised mean (SD)						
Total	38.3 (7.5)	42.0 (5.4)	0.001	27.1 (8.8)	36.6 (8.4)	0.001
Bulbar	8.6 (2.6)	11.6 (0.7)	0.001	6.8 (3.5)	11.4 (1.6)	0.001
Spinal	18.8 (6.4)	18.8 (5.1)	ns	10.9 (7.0)	14.4 (6.6)	0.02
Respiratory	11.0 (1.3)	11.6 (0.9)	0.002	9.4 (2.2)	10.9 (2.2)	0.002
Forced vital capacity mean (SD)	63.8 (21.9)	85.2 (17.9)	0.001	37.3 (31.5)	60.0 (29.4)	0.001
Laryngeal adductor reflex *N* (%)						
Present	52 (61.2)	58 (96.6)	0.001	48 (40.0)	22 (88.0)	0.001
Poor	25 (29.4)	1 (1.7)		30 (25.0)	2 (8.0)	
Absent	8 (9.4)	1 (1.7)		42 (35.0)	1 (4.0)	

*Data are expressed as mean (±SD) or frequencies [N = number (% = percentage value)]*.

**Figure 1:**
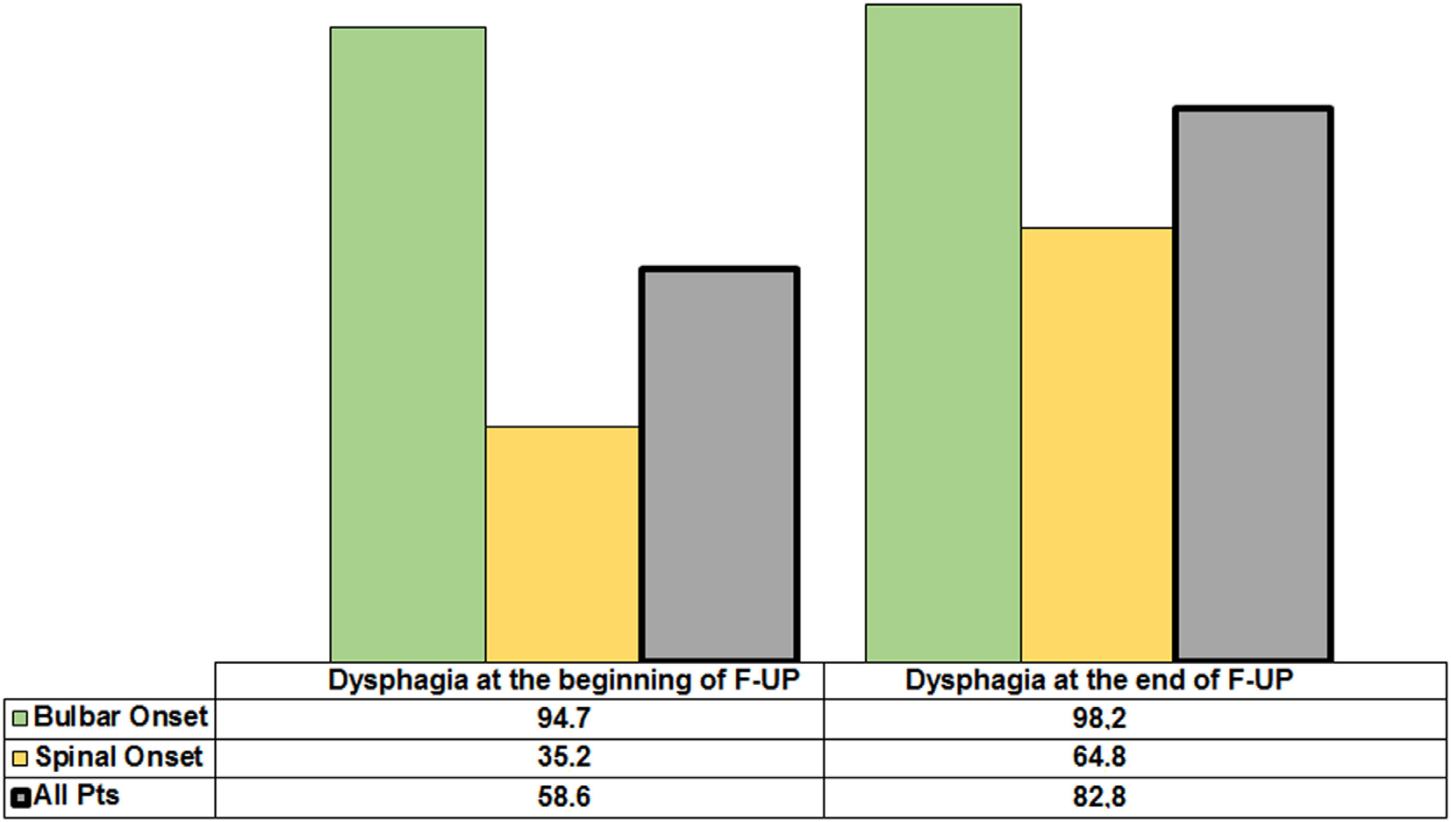
**Incidence of dysphagia in the whole amyotrophic lateral sclerosis population and according to spinal or bulbar onset at the beginning and the end of follow-up**.

**Figure 2:**
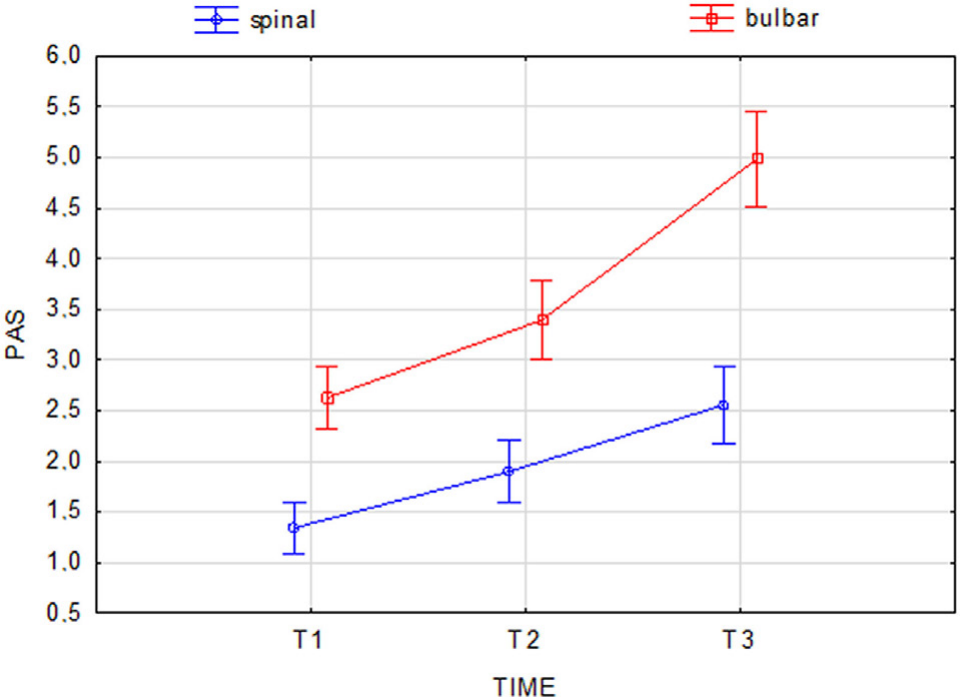
**Change in Penetration Aspiration Scale (PAS) score during the follow-up period in spinal and bulbar patients**. Current effect: *F*(2.284) = 15.210, *p* = 0.001. Vertical bars denote 0.95 confidence intervals.

**Figure 3:**
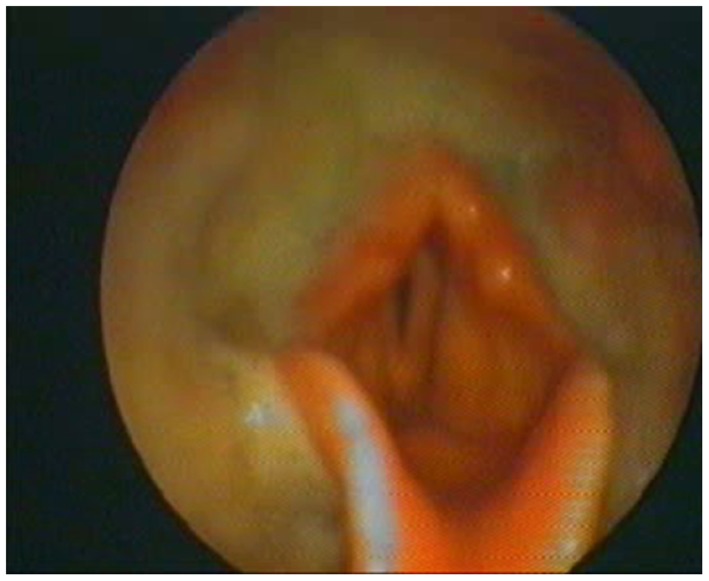
**The fiberoptic endoscopic evaluation of swallowing of one amyotrophic lateral sclerosis patient who did not perceive the objectified disorder but with abundant hypopharyngeal residues**.

At the beginning and at the end of the follow-up period, dysphagia was present in 58.6% (85/145) and 82.8% (120/145) of patients, respectively, with higher frequency in bulbar than in spinal patients (Figure 1). A delayed diagnosis of 14.2 and 18 months was found in dysphagic and non-dysphagic patients, respectively. Abnormal LAR and lower final FVC (*F* = 53.23, η^2^ = 0.27, *p* = 0.001) were more frequent among patients with bulbar onset. As expected in a progressive disease, a gradual significant worsening in PAS score during the follow-up period was observed (Figure 2). Six patients (8%) with dysphagia found by FEES did not perceive the disorder (Figure 3).

Fifty-four patients died (35% bulbar, 65% spinal) during the follow-up, while nine patients survived positioning tracheostomy. At the end of follow-up, disease duration was lower in bulbar onset patients than in those with spinal onset.

Sixty-three patients were deemed to have a poor prognosis (54 patients plus 9 who underwent tracheostomy). They showed a significant lower FVC (32.7 + 27.3 vs. 47.7 + 34.2, *p* = 0.004) in comparison to patients who survived. Fifty-five (87%) patients were dysphagic, and 25% of them refused the suggested thickening agents.

Percutaneous endoscopic gastrostomy was applied 30.6 ± 19.8 months after the onset of symptoms. Also, 29% of patients with PEG died, showing, however, a longer mean disease duration (38.0 ± 21.0 months) when PEG was positioned earlier (28.7 ± 22.4 months).

### Diet Assessment

At the beginning of the observation period 63 patients were on a normal diet, 64 on a soft diet, 13 on a pudding diet, and 5 on a puree diet. No patient used thickening agents or needed PEG. At the end of the follow-up, the following diets were adopted: normal in 24 patients, soft in 45 patients, pudding in 35 patients, and puree in 11 patients; 30 patients underwent a PEG. Bulbar dysphagic patients were given foods with modified consistency and underwent PEG more frequently than those with spinal onset (*p* = 0.01).

A detailed description of liquid intake is reported in Table 3. Only 56% (30/54) of patients used the suggested thickening agents. In particular, 49% of dysphagic patients with spinal onset refused the proposed thickeners, while only 15% with bulbar onset refused them (*p* = 0.001 for the difference between bulbar and spinal ALS patients). Fifteen patients (62%) who refused the proposed thickeners died, while three survived after placing PEG.

**Table 3:** **Description of the liquid intake in dysphagic patients (entire population and subgrouped by onset)**.

Intake	All dysphagic patients (*N* = 120)	Spinal onset dysphagic patients (*N* = 64)	Bulbar onset dysphagic patients (*N* = 56)	*p*
Normal	8 (7)	7 (11)	1 (2)	ns
Postural	31 (26)	20 (31)	11 (20)	ns
Syrup/nectar	13 (11)	12 (19)	1(2)	0.007
Cream	43 (36)	22 (34)	21 (37)	ns
Percutaneous endoscopic gastrostomy	25 (20)	3 (5)	22 (39)	0.0001

*Data are expressed as frequencies [numbers (%)]*.

*Normal; postural, tuck chin; density 1, syrup (nectar) 51–350 mPa s can be drunk with a straw or a cup if recommended; it leaves a thin veneer on the back of the spoon; and density 2, cream 351–1,750 mPa s cannot be drunk with a straw; it can be drunk from a cup; leaves a thin veneer on the back of the spoon*.

Only 75% of patients with poor or absent LAR used thickeners, diets with modified consistence, or PEG. Twelve (63%) of the patients who refused the proposed thickeners died.

### Riluzole Management

**Figure 4:**
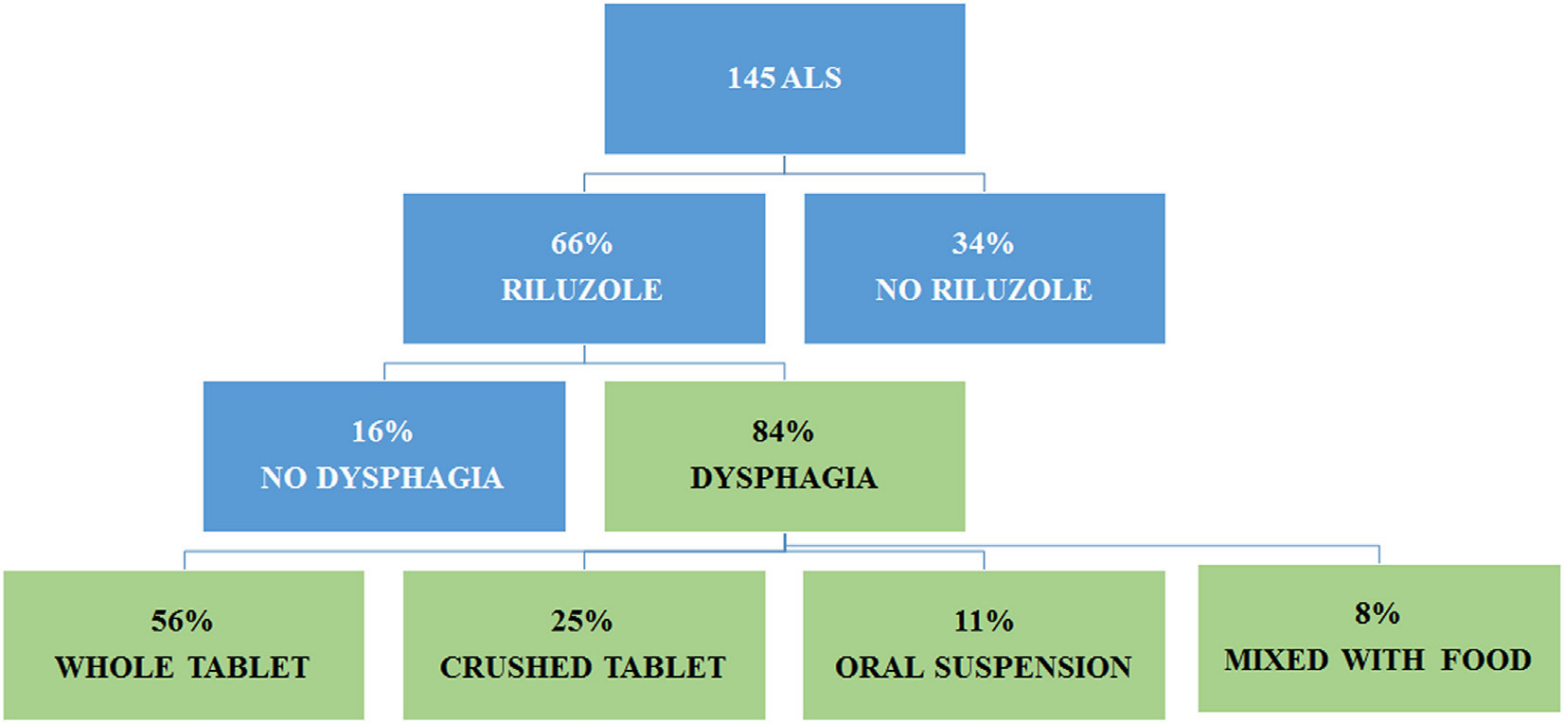
**Administration of riluzole in amyotrophic lateral sclerosis (ALS) patients according to dysphagia**.

At the end of the follow-up period, 95 patients were on riluzole, while 50 patients did not take the drug because of excessive sedation, increase of levels of transaminases, or patient refusal. Among the treated patients, 80 had dysphagia. They took the drug as follows: 51 patients used whole tablets (45 with water, 3 with fruit juice, 2 with yogurt, and 1 with fruit or bread), 20 crushed the tablets (7 with a spoon and 13 with a hackneyed tablets), and 9 assumed the oral suspension (Teglutik^®^, available on the market since March 2013) (Figure 4).

Among the 20 patients crushing riluzole tablets, 17 mixed them with water (16 through PEG, 1 to swallow), 1 mixed with tea, 1 with fruit, and 1 with a thickening solution.

The use of riluzole did not impact on survival either in dysphagic or in non-dysphagic patients (Gehan’s Wilcoxon test = 0.07; *p* = 0.93). In dysphagic patients, no difference in the time of death was observed between patients with bulbar and spinal onset (Gehan’s: Wilcoxon test = −1.23; *p* = 0.21).

Forty-eight percent of patients untreated with riluzole died, compared to 45% of those treated with whole tablets, 35% with crushed riluzole, and 22% with riluzole oral suspension. A significant difference in terms of mortality was found between patients untreated with riluzole and those treated with riluzole oral suspension (*p* < 0.05).

## Discussion

In this study, we evaluated the clinical features associated with dysphagia in ALS patients and their impact on riluzole and food management.

Our data are consistent with the literature: at the beginning of the disease, dysphagia was prevalent in patients with bulbar onset, and the duration of the disease affected mostly the frequency of dysphagia in spinal patients (21). Swallowing problems are often underestimated in ALS patients due to the progressive adaptation to slow deterioration of bulbar function, and the patient does not always recognize the disorder, leading to serious consequences and decreased survival. Dysphagia needs to be treated early and regularly to prevent complications, particularly in patients who do not initially perceive the disorder. This ensures adequate therapy through prevention of food aspiration and support of oral alimentation (*via* compensatory postures and dietary modifications), postponing PEG (22, 23). In the present study, 8% of spinal patients presenting with signs of dysphagia by FEES did not report the disorder as symptomatic. These findings emphasize the importance of performing a periodic objective swallowing evaluation in all ALS patients. Strategies to prevent malnutrition have a positive impact also on survival and quality of life (24, 25).

Recently, an important role has also been attributed to laryngeal sensitivity in the assessment of neurogenic oropharyngeal dysphagia (26). Laryngeal sensitivity is crucial for maintaining safe swallowing, thus avoiding silent aspiration. Until now, pharyngo-laryngeal sensitivity has received limited attention in patients with ALS, mainly because of the belief that ALS spares sensory function (27). In our study, FEES and PAS were able to objectively assess changes in the swallowing function over time, and this was particularly relevant in spinal patients, where the dysphagia may not even be recognized.

In patients with silent dysphagia, early detection is critical to avoid complications such as pneumonia. The management of oropharyngeal secretion and the efficacy of cleaning mechanisms, such as coughing and throat clearing, can be assessed simply and directly by FEES (28). Moreover, it can be performed at the bedside, thus facilitating the examination of severely motor-impaired, bedridden, or uncooperative patients. The AAN practice parameters and European ALS Consortium suggests that nutrition education and management is the standard of care and recommends regular nutrition evaluation every 3 months in ALS patients (29). Treatment of dysphagia is based on swallowing techniques, which improve the patient swallowing abilities thus reducing aspiration risks, and on diet change to adapt the consistency of foods and liquids to the patient specific impairment (30). The multidisciplinary approach has improved the overall care of ALS patients. Nonetheless, otolaryngologists are often not included in ALS management until a tracheostomy is considered. In our series, 44% of dysphagic patients, above all dysphagic patients with spinal onset, refused requested thickeners or PEG, and 25% of them had an impaired LAR. Paradoxically, we noted that patients who accepted thickening agents worsened more frequently compared to patients who refused them (67 vs. 47%). An accurate analysis of data, however, showed that non-compliant patients had a low level of disability and a better lung capacity. This suggests that any person who considers himself really ill makes everything possible to survive.

Seventy percent of patients who used PEG survived, and these data confirm that early recognition of dysphagia and of different grades of aspiration into the airways allows to identify patients who need PEG early on (29, 31, 32).

According to the literature, patients who were not treated with riluzole died more frequently compared to those receiving the drug, even if a significant difference was noted only for patients treated with oral suspension of riluzole (33). Since swallowing a pill is particularly problematic in these patients, the riluzole tablet is often crushed and dispensed with food. Both practices are not in line with riluzole label instruction, and no data are available about the efficacy and safety of crushed riluzole tablets. It is also important to remember that riluzole has anesthetic effects, as it partially blocks sodium channels, and it is usually administered in coated tablets to avoid oral paresthesia (34, 35). Considering the possible larynx sensory deficit in dysphagic ALS patients, the practice of crushing riluzole tablets could impair the swallowing ability, therefore increasing the risk of aspiration. Moreover, this potential anesthetic effect could be prolonged if riluzole is dispersed in food, given the long time required by ALS patients to finish a meal. A comparative study between whole, crushed, and oral suspension of riluzole on a large number of ALS patients may help to understand the possible conditioning effect of different absorption of riluzole. Actually, the oral suspension of riluzole allows patients who suffer from dysphagia to receive the only approved ALS treatment in a way that does not alter the drug and is in line with the riluzole dosing instructions. Moreover, the viscosity of riluzole oral suspension is 300–700 cP (European Teglutik Patent No. 2405890B1), therefore similar to nectar and honey, allowing to minimize the risk of aspiration.

In conclusion, dysphagia is a very common symptom in ALS patients. The disease duration influences the occurrence of dysphagia especially in patients with spinal onset, appearing instead very early in patients with bulbar onset. Patients adapt food consistency according to the worsening of dysphagia, and they also alter riluzole tablet to swallow the drug. The pureed diets and PEG prescription increased over time, even if a high percentage of patients refused requested thickeners or PEG, despite LAR impairment. The oral suspension of riluzole would allow the safe administration of the drug in ALS patients with dysphagia without the need to crush the tablet or disperse it in food, practices that are inconsistent with their safe and effective use. An early and periodic nutrition assessment and therapeutic intervention is necessary to adequately modify the consistency of the diet and the proper use of thickening agents or PEG.

## Author Contributions

EO and MI: design of the study, interpretation of the data, preparation, and approval of the manuscript. EO, GR, IS, MG, and MI: analysis and interpretation of the data, preparation, review, and approval of the manuscript. IS, VF, MC, and CC: collection and interpretation of the data as well as review and approval of the manuscript.

## Conflict of Interest Statement

The authors declare that the research was conducted in the absence of any commercial or financial relationships that could be construed as a potential conflict of interest.
